# Efficacy and efficiency of indoor nighttime human external cargo mission simulation in a high-fidelity training Centre

**DOI:** 10.1186/s13049-020-00755-4

**Published:** 2020-06-29

**Authors:** Urs Pietsch, Volker Lischke, Stephen J. M. Sollid, Stephan Prückner, Lorenz Theiler, Robert Greif, Roland Albrecht

**Affiliations:** 1 Department of Anaesthesiology and Intensive Care Medicine, Cantonal Hospital St. Gallen, St. Gallen, Switzerland; 2 Air Zermatt, Emergency Medical Service, Zermatt, Switzerland; 3 Bergwacht Schwarzwald, Hessen, Bayern Germany; 4 Norwegian Air Ambulance Foundation, Oslo, Norway; 5 University of Stavanger, Faculty of Health Sciences, Stavanger, Norway; 6 Institut für Notfallmedizin und Medizinmanagement, Klinikum der Universität München, Ludwig-Maximilians-Universität München, Munich, Germany; 7 Department of Anaesthesiology and Pain Medicine, Inselspital, Bern University Hospital, University of Bern, Bern, Switzerland; 8 School of Medicine, Sigmund Freud University Vienna, Vienna, Austria; 9 Swiss Air-Rescue, Zurich, Rega Switzerland

**Keywords:** HEMS, Human external cargo, Training, Patient safety, Night mission

## Abstract

**Background:**

The human external cargo (HEC) operations conducted by Helicopter Emergency Medical Services (HEMS) rarely take place at night, making it difficult for crew members to attain and maintain the level of expertise needed to perform winch operations in the dark. As EASA requirements for training cannot currently be met, we evaluated whether simulation training could be an option.

**Methods:**

This paper reports on a training concept using indoor simulation for the training of nighttime HEC operations. Participants’ experience and perceptions were evaluated with a survey and the procedural and economic advantages of the simulation approach were compared with those of the usual outdoor HEC training.

**Results:**

Most participants had limited exposure to real-life nighttime HEC missions before undergoing the simulation-based training. The frequency of training cycles in simulation was much higher compared to conventional training (60 cycles indoors vs. 20 outdoors for HEMS-TC, 20 cycles indoors vs. 4 outdoors for MCM). Trainees perceived that their technical and non-technical skills (NTS) improved with the training. The estimated costs of standard outdoor-based nighttime HEC training (138€ per cycle) are at least 6.5 times higher than the costs of indoor simulated training (approximately 21€ per cycle). With a change to simulation, carbon dioxide emissions could potentially be reduced by more than 35 tons.

**Conclusions:**

Indoor simulation training of night HEC operations has advantages with regard to cost-effectiveness, environmental friendliness, and self-reported improvements in skills and knowledge. Its use is feasible and could improve crew and patient safety and fulfill regulatory demands for training intensity.

## Background

Training is a key factor in the performance of members of the Helicopter Emergency Medical Services (HEMS) in Alpine emergency situations. Human external cargo (HEC) missions, and in particular winch operations, are routine, but HEC missions occurring in the night and darkness are rare.

The Bavarian Alpine rescue service runs an internationally recognised mountain rescue training centre with the capability of simulating HEMS winch operations. Because live, in-flight helicopter training is cost-intensive, logistically challenging, and environmentally unfriendly, high-fidelity indoor simulation could be a promising alternative.

The European Union Aviation Safety Agency (EASA) [1] requires initial and recurring training within a 12-month cycle for HEC maneuvers. This training uses significant resources and is costly due to the required flight time and staff costs.

Simulation training applying crisis resource management (CRM) principles aims to address human factors (HF) in aviation and medicine [2, 3]. As a considerable number of errors are caused by HF, simulation training provides an opportunity to manage complex medical situations under standardised conditions without endangering patients, thus reducing the occurrence of errors and increasing crew and patient safety.

Previous studies [4–9] describing the use of simulation training in HEMS show an increase in self-reported confidence among crew members [6, 9].

Unpublished data show that a typical medical crew member (MCM) at Swiss Air-Rescue (Rega) or Air Zermatt has less than three HEC missions at night in 10 years. The low number of missions and their complexity highlights the need for training at night.

In 2010, the German federal mountain rescue services (Bergwacht) developed a joint education concept for mountain HEMS operations, including a dedicated simulation facility [6]. However, the effect of simulation training on rarely performed HEC operations has not been studied.

Our study aims to determine whether nighttime indoor simulation training is more effective and efficient than conventional live nighttime training in terms of cost-effectiveness, environmental friendliness, and self-reported improvements in skills and knowledge.

## Methods

In a prospective observational study we evaluated the use of an indoor HEC night simulation program for the training of nighttime winch operations by the Swiss HEMS. We used a questionnaire to evaluate course participants’ reactions to the training, we estimated the costs of training (personnel, equipment maintenance, etc.) and we calculated the potential reduction in CO2 produced with traditional training. Indoor simulation was compared with traditional outdoor HEC performed by the Swiss Air-Rescue (Rega) and Air Zermatt (Switzerland).

### Indoor simulation of night HEC missions

Members of the instructor team (HEMS emergency physicians, paramedics, and rescue specialists with experience in HEC rescues) from the Mountain Rescue Centre Bad Tölz, Bavaria, Germany (Bergwacht Zentrum für Sicherheit und Ausbildung [BWZSA]) developed the simulation course based on Air Zermatt’s current standard operating procedures (SOPs) (Table 1).

**Table 1:** Course curriculum

**Day 1** Focus on HEC in the darkness Safety first. Even in a simulation, there are real risks and hazards. During the training, the maximal fall could be 25 m.	**Classroom** Team resource management and HF HF and CRM refresher Task management Teamwork and leadership Situational awareness Decision making Medical topics (e.g. ACLS of a hypothermic patient; simple and advanced trauma treatment) Safety briefing **Simulator** Safety briefing Six different simulated scenarios focusing on safe HEC in the darkness. Scenarios (increasing in complexity): - Preparing a patient (recumbent in a rescue bag or sitting) for an HEC rescue in “safe” terrain. - Picking up in a steep wall with self-belaying in combination with or without a patient (recumbent and sitting)
**Day 2** Focus on consolidating knowledge and skills	**Simulator** Five different simulated scenarios and debriefings Focus: Onsite interventions and treatment in challenging terrain, with reduced personnel resources, and limited monitoring.
**Day 3** Transfer of what has been learned into future daily clinical practice	**Classroom** Moderated discussion involving all participants, instructors, and the medical director, on what has been learned

The simulation took place at the BWZSA, which houses two specially developed three-axis cranes that suspend two full-scale mock-up helicopter fuselages (one decommissioned Eurocopter BK117 and one Super Puma-sized mock-up), simulating flying at about 20 m altitude. The helicopter fuselage has a rescue hoist with a double cargo hook, allowing standardised training of the full range of HEC manoeuvres. A suspended cableway, with a chairlift and a gondola lift, along with various “terrain structures” (e.g., a boulder, a rock ridge, a cave and uneven surfaces) allows for a wide range of alpine close-to-reality scenarios (Table 2).

**Table 2:**
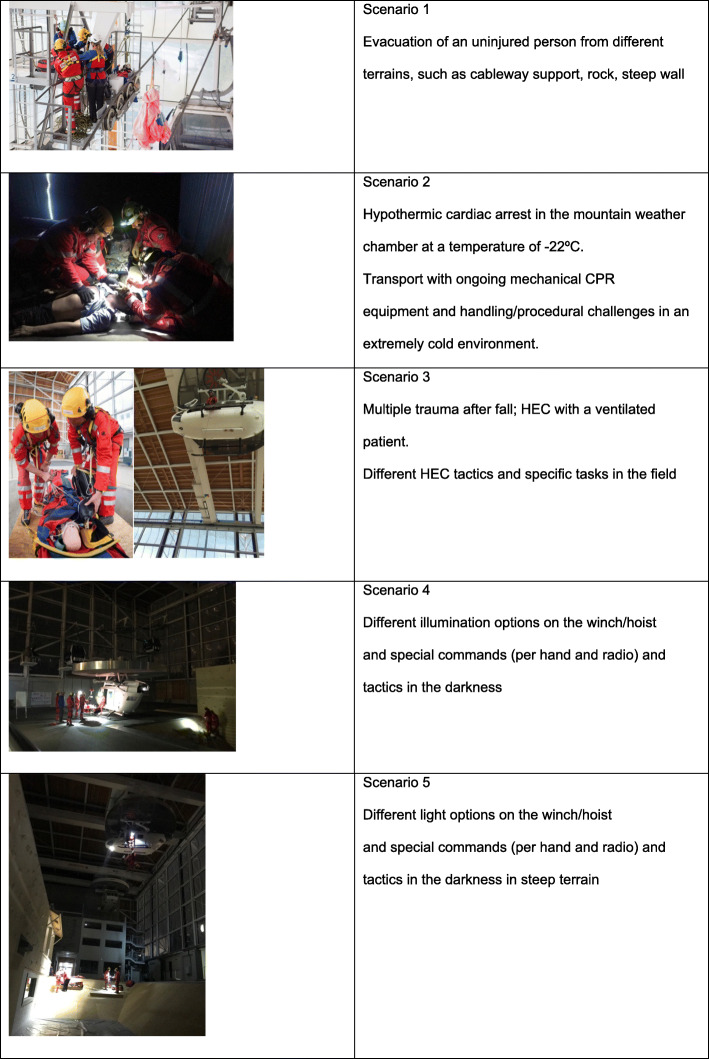
examples of the simulated scenarios

The simulation training started with a didactic classroom refresher on HEC manoeuvres, focusing on different times (day vs. night), mountain rescue performance (typical mountain-related medical emergencies such as multiple trauma, hypothermia and evacuation of an uninjured person), and HFs, NTS and CRM (special commands per hand and radio, light options on the winch/hoist in the darkness, etc.). Each participant took part in five different simulated scenarios.

Each simulation started with an initial briefing of the scenario, followed by the simulated mission and then a focused standard simulation debriefing by certified simulation instructors. The structured debriefing allowed team members to reflect on their skills performance and team communication. The debriefing also addressed procedural errors in the rescue that might have jeopardised safety.

At the end of each day of training, a moderated panel discussion involving all participants, instructors and the medical director summed up the lessons learned and experience to be transferred to daily practice.

### Study population

Six paramedics and 16 emergency physicians underwent simulation training over 2 days for 12 h per day. In the Swiss HEMS Crew configuration the paramedic—termed the HEMS-Technical Crew Member (HEMS-TC)—is the winch operator in the helicopter. The physician or medical crew member (MCM) is the one being hoisted down to the patient.

### Evaluation

The HEMS-TC and MCM rated the simulation training using an established [6] “Pre-Post-Training Self-Evaluation Questionnaire”. The questionnaire was designed to assess the training on a reaction and learning level [10]. This self-assessment questionnaire included three parts, with the first two filled out before the simulation training course. Part one included questions on demographics and the individuals’ experience in HEMS and HEC missions. Part two assessed textbook knowledge and NTS related to nighttime HEC missions. In part three, the questions from part two were repeated immediately after the simulation training course. The answers to multiple choice questions were given using the 6-point Likert scale (1 = best possible; 6 = worst possible. Knowledge questions were answered with free text.

Numbers of winch cycles during the indoor simulation and usual outdoor training were recorded and compared. The logistical and personnel costs of the indoor simulation training course were calculated and compared to the cost of traditional outdoor helicopter HEC training conducted by Swiss Air-Rescue (Rega) and Air Zermatt.

## Results

### Participants’ experience

Twenty-two questionnaires (100%, 6 HEMS-TC, 16 MCM) were analysed. All MCMs were consultant anaesthesiologists, with a mean of 16.3 (± 6.2 SD) years of prehospital emergency medicine clinical practice. Two paramedics (senior hoist instructors) were certified flight paramedics and critical care paramedics; four were paramedics in training (less than 2 years of HEMS expertise).

Fourteen of 16 MCMs (88%) had extensive experience in real-life daytime HEC missions (> 50), but none of them had more than 50 HEC missions at night (Table 3). Experience ranged between 10 and 50 missions. Four HEMS-TCs (67%).

**Table 3:** Expertise in daytime and nighttime HEC missions (real-life missions)

Number of HEC missions	HEMS TC night	HEMS TC day	MCM night	MCM day
**0**	1	0	2	0
**< 10**	0	0	6	0
**10–50**	5	2	8	2
**> 50**	0	4	0	14

had > 50 daytime HEC missions and five HEMS-TCs has fewer than 50 nighttime HEC Missions (Table 3).

In the usual outdoor training, an MCM goes through four winch/hoist practice cycles and an HEMS-TC goes through 20 cycles (Table 4). In comparison, in indoor simulation training, an MCM went through 20 winch cycles and the HEMS-TC went through 60 cycles.

**Table 4:** Number of winch/hoist cycles during the training (per person)

	Outdoor “usual” curriculum	Indoor simulation
HEMS TC (Winch operator)	20	60
MCM	4	20

Textbook knowledge and CRM questions improved in all domains between the pre-course and post-course questionnaire (Table 5).

**Table 5:** Pre-Post-training evaluation questionnaire (Likert scale; 1 = most positive score possible, 6 = worst possible)

I estimate my ability to …	Before Training Mean (SD)	After Training Mean (SD)
... keep track in a HEC mission in **darkness**	3.1 (0.61)	1.9 (0.57)
... set the right priorities and perform a safe HEC rescue in the **darkness**	2.4 (0.76)	1.8 (0.65)
... set the right priorities and perform a safe HEC rescue in the **daytime**	2.7 (0.81)	2.0 (0.71)
... consider all available information and all available resources during an HEC Mission in the **darkness**	3.2 (0.76)	2.1 (0.70)
… communicate efficiently in an HEC mission in the **darkness**	2.6 (0.61)	1.9 (0.65)

### Cost comparisons and cost savings

To compare the efficiency of the simulations and standard outdoor training, information was collected regarding the costs of personnel (attendees and instructors), helicopter and winch maintenance, fuel expenditures, and the use of the simulation center. Winch maintenance for an Airbus H145 helicopter was required after every 100 winch cycles. The cost of an indoor HEC cycle was calculated at approximately 21€ per cycle, compared with 138€ per standard outdoor HEC cycle (Table 6)

**Table 6:** Costs

	Cost per HEC cycle
**Outdoor traditional helicopter HEC training**	138€ Flight 50€/min, per HEC cycle, ca. 2-3 min.
**Indoor simulation**	21€ (7750€/350 HEC cycles)

.

### CO2 production

Production of carbon dioxide emissions was difficult to measure precisely. We estimate that 1 h of outdoor training uses about 260 kg of fuel and thus generates nearly 780 kg CO2. In more than 45 h per year of real flight time for required outdoor training, this would be a savings of more than 35 tons of CO_2_ if HEC training were held indoors.

## Discussion

Our study revealed that structured indoor HEC training for night HEMS missions is both effective and efficient. Self-assessed NTS and textbook knowledge of HEC missions improved among all participants. The costs of standard outdoor-based nighttime HEC training—including follow-up expenditures for helicopter and winch maintenance—are at least 6.5 times higher than the costs of indoor simulated training. In addition, indoor training provides participants with significantly more opportunities to perform winch cycles. Additional positive aspects of indoor training may include irrelevance of the weather, better environmental friendliness, and increased participant safety, although these were not measured in our study and are difficult to quantify.

### The benefit of night-time HEC simulation training

Based on our daily work experience and the analyzed data, we know that night HEC missions are approximately 10 times less frequent than day HEC missions [11]. An average HEMS physician in Switzerland has participated in fewer than three night HEC missions in 10 years (unpublished data). It is unlikely that this limited experience sufficiently prepares HEMS for safe performance of a complex intervention. This emphasizes the need for a training curriculum that can compensate for low real-time exposure.

### Existing training options

To our knowledge, no standardised guidelines or training programs for HEC missions—particularly for night HEC missions—are available. Many countries have mandatory training programs for rescue and flight operative procedures, including recurrent flight simulator training for pilots and HEMS-TCs. Medical training, simulation-based or otherwise, depends on local initiative and commitment in most countries.

There are a few simulation programs in HEMS that focus on issues like medical skills, NTS or CRM [4, 6, 7, 9]. A study from the Norwegian HEMS showed that HEMS personnel lack simulation-based training and assessment of their NTS [12]. Various studies have demonstrated that HEMS personnel report a significant increase in self-assessed competency of NTS after simulation training [4, 5, 12]. These NTS are essential to complement the technical skills needed in an intricate work setting such as mountain HEMS. Training in technical and non-technical skills needs to be tailored to mission profiles and to procedures like HEC.

Our working group established an indoor medical simulation training course (Med-Sim BWZSA) tailored to the needs of mountain HEMS [5, 6]. This course has since become mandatory for all mountain rescue physicians working in cooperation with the Bergwacht, Germany.

### Curriculum

The curriculum presented in this study was tailored to the needs of mountain HEMS performing rescues at night. We were able to demonstrate that confidence in their NTS and technical skills increased in all participants. The high number of winch cycles—more than 60 for the HEMS-TC and 20 for the MCM—provides a level of experience in night HEC far beyond the life-time exposure to real-life night HEC missions. During indoor simulation it is possible to train and supervise standard and emergency procedures, such as radio failure, fixed winch-cable and abortion of an operation — in a safe environment with minimal risk to the helicopter or crew.

### Economic aspects

Not only do training flights generate costs due to flight time, but there are also follow-up costs, such as maintenance of the helicopter and winch. We estimate that our indoor simulation training curriculum could minimize the costs of training by a factor of 6.5. Additionally, it could contribute to a relevant reduction in fuel, noise emissions, and CO_2_.

Proving the economic benefits of simulation training is challenging, however. To our knowledge, only one study has compared the costs of HEMS simulation and real-life training [9]. Dotson et al. found no statistical significance in the number of orientation flights between a simulation training group and a standard group [9], but there was a trend toward decreasing the cost of training with a high-fidelity air medical simulator.

### Maintaining safety standards

Night HEC missions represent less than 10% of all HEC missions in Switzerland. This makes it impossible to attain and maintain a high level of expertise in real rescue missions. Until now, training outdoors with real flights has been the only way to fulfil EASA requirements. Ensuring that the skills required for HEC remain current is associated with considerable financial and training burdens.

### Fuel consumption

The fuel consumption for 1 h of flight (no prolonged or additional hover operation) for an EC135 with Arrius 2B2 engines is nearly 260 kg/h. The CO_2_ emitted by burning jet fuel is approximately 3 kg per kg jet fuel. So in 1 h of indoor training it can be estimated that about 260 kg of fuel and nearly 780 kg of CO_2_ can be saved.

This could potentially reduce CO_2_ emissions by more than 35 tons for the entire service when the output deriving from the annual HEC flight training requirements is taken into account.

### Finding the ideal balance

Indoor training provides a safe environment that is not limited by bad weather conditions. It also allows for long-term planning, which is especially useful for part-time crew members who have other job obligations besides HEMS. With in-door simulation it is potentially possible to train night missions during regular working hours, thus further reducing personnel costs.

Indoor simulation cannot completely replace outdoor training, however. Some important aspects of HEC missions—such as dynamic winching—are difficult to train in an in-door simulation setting, as are the effects of noise, altitude and adverse environmental conditions.

It is important to find the ideal balance between indoor simulation and real outdoor training. This should be based on the needs of the HEMS provider and on the experience level of the staff. For a beginner attending HEC education for the first time, and for the annual required refresher course, indoor simulation seems to be an ideal choice.

### Limitations

Our study has some limitations. First, the pre-post questionnaire was not validated. However, it has previously been used in other simulation courses [6], and is based on the “Best practice and sample questions for course evaluation surveys” of the University of Wisconsin [13]*.* Although it is believed that NTS training benefits patient safety, the evidence supporting this thesis is still limited. Simulation-based team training seems to be the most prominent mode of training in the literature. But few objective data have been collected on this topic, and most results are subjective, based on trainees’ responses to questionnaires, as in this study [6, 9].

Second, even if the indoor simulator setting is real, it is not real life. Some aspects of mountain rescues—such as downwash—cannot be simulated. We do, however, think that these drawbacks are acceptable given the main focus and advantages of this HEC simulation.

Third, the current simulation course focuses predominantly on the MCM being winched and the HEMS-TC serving as winch operator; it does not replace the training of the winch operator and pilot together. A possible next step could be to focus on the whole team, perhaps also including pilots in training.

## Conclusion

Indoor simulation training of night HEC operations has advantages with regard to cost-effectiveness, environmental friendliness, and self-reported improvements in skills and knowledge. Its use is feasible and it could improve crew and patient safety and fulfill regulatory demands for training intensity.

The potential reduction in fuel consumption, CO_2_ emissions and noise compared to real helicopter flights are strong arguments for indoor human external cargo simulation. Future studies should evaluate the effect such training has on relevant outcome parameters in real missions.

## Data Availability

Not applicable.

## Abbreviations

EASA: European Union Aviation Safety Agency
HE: Human Error
HEMS: Helicopter emergency medical service
HEMS-TC: HEMS-Technical Crew Member
HCS: Human cargo sling
HEC: Human external cargo
HF: Human factors
HHO: Helicopter hoist operation
IQR: Interquartile range
MCM: Medical crew member
NTS: Non-technical skills
SOP: Standard operating procedure
